# Engineered Nanoparticles in Consumer Products: Understanding a New Ingredient

**DOI:** 10.1289/ehp.119-a120

**Published:** 2011-03

**Authors:** Rebecca Kessler

**Affiliations:** **Rebecca Kessler**, based in Providence, RI, writes about science and the environment for various publications. She is a member of the National Association of Science Writers and the Society of Environmental Journalists

In October 2010 the National Organic Standards Board recommended that engineered nanomaterials (ENMs) be prohibited from food products bearing the U.S. Department of Agriculture’s coveted Organic label.^1^ If the department adopts the recommendation, ENMs will find themselves in the same officially taboo category as genetically modified organisms when it comes to organic foods—nanotechnology-enabled innovations like flavor- and texture- enhancing ingredients and shelf life– extending packaging will be off the menu.

Prior to issuing its recommendation, the board received thousands of public comments and petition signatures supporting the ban and virtually none opposing it. Although an official decision could take years, supporters are confident the recommendation will be adopted, and it will go down as one of the first lines drawn in the sand when it comes to the reach of this relatively new and potentially transformative technology in the American marketplace.

Nanotechnology-enabled products are quietly proliferating on U.S. store shelves, despite nagging questions about the safety of synthetic nanoparticles and the products that contain them. “[I]n our regulation of food and most consumer products, we don’t implement the precautionary principle. Things go to market before we know whether or not they’re really safe for human beings over the long term,” says Alexis Baden-Mayer, a lawyer with the Organic Consumers Association, an advocacy group, who attended the meeting and campaigned for the ban.

Baden-Mayer and other observers perceive a distinct lack of public awareness about how common ENMs are becoming in the market-place, and she hopes discussion among consumers of organic products will help change that. “Consumers don’t know much about nanotechnology, and the first time they may hear about it is now when they learn that the organic regulations are going to prohibit [it],” she says.

The International Organization for Standardization defines a nanomaterial as a material with any external dimension between 1 and 100 nm.^2^ (By comparison, a double strand of DNA is about 2 nm thick.) Nanoparticles, which have been the focus of most nanotoxicology studies to date,^3^ are one subset of nanomaterials. Nanoparticles include structures of various shapes, such as nanotubes, nanowires, quantum dots, and fullerenes. They also occur naturally in substances like air, smoke, and sea spray, and “incidental” nanoparticles are created during processes such as combustion and food milling, churning, freezing, and homogenization. (Naturally occurring and incidental nanoparticles were not included in the National Organic Standards Board’s recommendation to ban ENMs.)

Nanotechnology—the deliberate synthesis and manipulation of nanomaterials—began in the 1980s. Today thousands of ENMs are manufactured in a kaleidoscope of substances, shapes, and sizes for use in a wide range of products and industrial processes that take advantage of their novel physical, thermal, optical, and biological properties. These properties may be determined by the ENM’s chemical composition, size or shape, crystal structure, solubility, adhesion (the force that holds the nanoparticle components together), or surface chemistry, charge, or area.^3^

Industry analysts have been forecasting “game-changing” advances as a result of nanotechnology in renewable energy, computers, communications, pollution cleanup, agriculture, medicine, and more.^4^ Clothing, sunscreens, cosmetics, sporting equipment, batteries, food packaging, dietary supplements, and electronics are just a few of the types of nanotechnology-enabled goods in use by U.S. consumers.

But safety questions arise around the nanoparticles in some of these products. The novel biological and physical properties of some ENMs pose unique challenges to comprehensive safety research, and investigators are working to figure out just how hazardous they might be to people, wildlife, and the environment. Compared with larger particles, nanoparticles’ tiny size means tissues may take them up more readily. It also can give them an unusual ability to travel throughout the body, including into cells and cell nuclei, and across the placenta and the blood–brain barrier, as demonstrated in rodent studies.^5^,^6^

No cases of human illness or death have been definitively attributed to ENMs. However, a number of researchers and consumer and environmental advocates have warned that the abundant unknowns make it necessary to proceed with caution lest we repeat the history of asbestos, polychlorinated biphenyls, the insecticide DDT, and other innovations that seemed valuable when they were introduced, proceeded with little oversight, and ultimately caused major health or environmental problems.

## What’s on Store Shelves?

As of 2007, the National Science Foundation estimated that up to $70 billion worth of nanotechnology-enabled products were sold in the United States annually, and that number is predicted to grow explosively.^7^ But pinpointing exactly which products contain ENMs is not always easy.

Manufacturers are not presently required to report the use of ENMs except for single-and multi-walled carbon nanotubes, for which the U.S. Environmental Protection Agency (EPA) finalized “significant new use” rules^8^ in September 2010.^9^ Another potential exception is the use of novel ingredients produced using nanotechnology in food or food packaging, which Sebastian Cianci, a U.S. Food and Drug Administration (FDA) spokesman, says “would in all likelihood require premarket approval.” However, a report last year by the U.S. Government Accountability Office concluded that “FDA’s approach to regulating nanotechnology allows engineered nanomaterials to enter the food supply as GRAS [generally recognized as safe] substances without FDA’s knowledge.”^10^

Manufacturers also are not required to label products containing ENMs, and there seems to be a recent trend toward dropping voluntary references to such ingredients from packaging, websites, and other publications, says Andrew Maynard, director of the Risk Science Center at the University of Michigan School of Public Health. In some cases, he notes, “manufacturers will either just use the name of the chemical without stating whether it’s at the nanoscale or not, or they’ll use words like ‘micronized,’ so it’s hard to work out whether it’s nanoscale.” The upshot, Maynard says, is that consumers are largely in the dark about whether the products they use contain ENMs.

In 2005 the Project on Emerging Nanotechnologies (PEN) at the Woodrow Wilson International Center for Scholars established an inventory of consumer products sold around the world that advertise having ENM content.^11^ The inventory now contains more than 1,000 entries.

David Rejeski, PEN’s director, says the inventory undoubtedly includes just a portion of the purportedly nanotechnology- enabled products on the market. Nonetheless, he says, the inventory has filled an important gap as the only catalog of its kind, and it has enabled PEN to pick up several important trends that might otherwise have gone undetected. One was the rise of nanosilver, often used as an antimicrobial agent in products such as odor-resistant garments and food storage containers designed to keep leftovers fresh longer. PEN also spotted distinct upticks in products designed for children and babies and in products manufactured in China and other Asian nations.

However, funding for the inventory— which came from the nonprofit Pew Charitable Trusts—has run out, and PEN has neither added to the database since August 2009 nor been able to convince another institution to adopt it. Rejeski says that without an up-to- date inventory, researchers and regulators have no way to track the products available to consumers or the kinds of ENMs they may be exposed to. “It’s not just a matter of the consumer not knowing,” he says. “It’s a matter of the government not knowing.”

## Products under Scrutiny

That kind of information is important because when it comes to ENMs, different products lead to different potential exposures and therefore pose different potential hazards. For consumers, experts say, the greatest exposure probably comes from products that are ingested or otherwise come into intimate contact with the body—things like dietary supplements, food, and personal care products.

The latter category includes such products as a hair growth–stimulating shampoo claiming to use copper nanoparticles,^12^ toothpastes with antibacterial silver nanoparticles,^13^ highend skin cream made with “energizing, detoxifying” gold nanoparticles,^14^ and “extreme wear” makeup.^15^ Sunscreens, however, have attracted the most attention from researchers and advocacy groups.

Many sunscreens include titanium dioxide or zinc oxide nanoparticles because they effectively block ultraviolet light while—unlike the thick white creams of yore—allowing the sunscreen to be transparent when it’s rubbed onto the skin, according to the Environmental Working Group (EWG), a public health and environmental advocacy organization. Testing of these products has focused on whether the nanomaterials can penetrate the skin, says Nigel Walker, deputy program director for science for the National Toxicology Program. As the saying goes, if there’s no exposure, there’s no risk, and several studies indicate that very little of the nanoparticles in sunscreen can penetrate the skin and enter the body—as long as the skin is healthy and intact.^16^

When it comes to sunscreens, Walker says, the research community is “pretty much comfortable that the amount of exposure for normal skin to nanoscale materials is extremely low, lower than many chemicals that we currently already use.” Even the EWG—which has petitioned the government to tighten regulation of nanomaterials in personal care products—concluded that the risk of ultraviolet radiation damage from not wearing sunscreen outweighs the risk of harm from nanoparticles.^17^

Still, concerns persist, especially since the lack of a labeling requirement means people with skin abrasions or rashes, which are possible exposure pathways, could have trouble avoiding ENM-containing sunscreens. And people, especially children, are prone to consuming small quantities of sunscreen accidentally when they rub it onto their faces and lips. A lot of sunscreen also washes off in natural waterbodies or runs down the drain when people shower. Although more research is needed, initial studies have shown that titanium dioxide and zinc oxide nanoparticles can harm algae,^18^,^19^ water fleas,^20^,^21^ and frogs,^22^ and that they can travel up the aquatic food chain with unknown environmental consequences.^23^

Titanium dioxide and zinc oxide ENMs, which are used in a huge array of products besides sunscreens,^24^ have been linked with potentially adverse health effects in some studies. For instance, a 2009 study from the University of California, Los Angeles, found that mice fed certain kinds of titanium dioxide nanoparticles with their drinking water for 5 days exhibited DNA and chromosomal damage and inflammation.^25^ In two separate studies the same year, a Japanese team showed that male offspring of pregnant mice injected with certain titanium dioxide nanoparticles experienced genital malformations and neurologic damage^6^ as well as changes in gene expression in the brain.^26^ Other *in vitro* studies have indicated some types of both titanium dioxide and zinc oxide nanoparticles are toxic to human brain and lung cells.^27^,^28^,^29^

ENMs in edible products have garnered less research attention, the recommendation to keep them out of organic foods not-withstanding. One reason may be that it is unclear to what degree foods containing ENMs have actually hit U.S. supermarkets, although many observers have noted that the field of food science is unmistakably abuzz pursuing nanoscale ingredients to improve texture, taste, nutrition, shelf life, and safety. Cianci acknowledges that some salad dressings and spreads now on store shelves may contain nanoscale oil droplets intended to slow the separation of ingredients and that some fruits and vegetables may carry an edible coating of nanoscale wax droplets. “These are arguably foods that have engineered nanomaterials in or on them but which raise no safety concerns compared to their traditional counterparts,” Cianci says.

As far as food packaging is concerned, the FDA is “not aware of any significant use of novel nanomaterials in the food packaging market at this time,” according to Cianci. However, reports by groups such as PEN,^30^ the conservation organization Friends of the Earth,^31^ and the British House of Lords^32^ indicate there is a great deal of research into using ENMs to develop advanced food packaging, and they point to several products already on the U.S. market, such as composite plastic bottles that incorporate nanoscale clays to extend the shelf life of beverages.

Dietary supplements are another market where nano is hot. The Source Vitamin Company, Inc., of Fort Lauderdale, Florida, for one, boasts of the All Natural Patented Nanotechnology™ driving its supplement products, which it maintains enables the targeted delivery and immediate absorption of active ingredients.^33^ How much truth there is behind such claims across the board is hard to assess because the FDA generally does not approve, test, or verify the labeling of supplements before they hit the market. Andrew Shao, senior vice president of scientific and regulatory affairs at the Council for Responsible Nutrition, a trade group, says many supplement manufacturers’ nano claims are simply “a marketing tactic.” And Daniel Fabricant, vice president of global government and scientific affairs at the Natural Products Association, another trade group, says he doesn’t know of any supplement manufacturers that are using nanoscale *dietary* ingredients— they are simply too expensive, he says, and their benefits are unproven.

At the same time, Shao says some supplements do contain nanoscale ingredients that facilitate manufacturing processes or enhance properties such as the clarity of liquids. He points out that no adverse effects related to nanomaterials in supplements have been reported to the FDA, adding “there is no evidence that there’s some kind of imminent threat as a result of the limited use and application of nanotechnology for supplements.”

Still, the inclusion troubles many researchers, in part because of the lack of any premarket approval by the FDA. More important, the health effects of ingesting ENMs remain poorly studied, researchers agree. “If you look at the data, virtually all of the tests [have] been done on exposure either through inhalation, through the skin, or through injection. There are virtually no studies on ingestion,” says Michael Hansen, a senior scientist with Consumers Union, which has pressed for tight regulation of nanoparticles in consumer products. Research into the effects of long-term exposure to ENMs also is sorely lacking, he and other experts say.

## Challenges to Health and Safety Research

Data on nanoparticle health and safety are hard won. Experts agree that many ENMs pose serious and unique scientific and methodological challenges to investigators, one very basic barrier being the ability to detect nanoparticles deposited in cells and tissues. For instance, it is exceedingly difficult to image materials smaller than 50 nm inside the human body, and quantifying carbon nanotubes is all but impossible, which poses a major challenge to assessing whether nanoparticles reach specific organs when evaluating data from toxicity studies. What instruments do exist can be prohibitively expensive, says Jaclyn Cañas, a toxicologist with the Institute of Environmental and Human Health at Texas Tech University who has been studying the environmental fate of carbon nanotubes.

And that’s only part of the problem. “It’s a huge challenge to do nanotox-related research not just from a financial standpoint [but] from an intellectual standpoint, trying to wrap your mind around it and then really come to the bottom line of what causes toxicity,” Cañas says. “It’s not as clear-cut as what we’re trained to do and the kinds of contaminants we’ve worked with before.”

Sheer numbers pose another basic but formidable challenge. Different coatings, sizes, surface charges, functionalizations, or manufacturing processes can drastically alter the toxicity and behavior of a given ENM, and companies are constantly developing new materials. By one estimate there are 50,000 different permutations of carbon nanotubes alone.^34^

Moreover, there can be a great deal of inconsistency between batches. In one 2007 study Maynard led a team in analyzing two batches of carbon nanotubes acquired from the same company.^35^ As is typical, the batches weren’t pure: they contained distinct mixtures of single and bundled carbon nanotubes, unstructured carbon, and other metal nanoparticles. When agitated, however, one batch released dense particles of about 100 nm that were composed mostly of unstructured carbon, whereas the other tended to release larger particles that were diffuse, spiderweb-like tangles of bundled carbon nanotubes.

“You can imagine that the two different types of particles would behave completely differently inside the lungs,” Maynard says. “So, notionally [they were] the same material, [but] in reality the types of particles being released were like night and day.”

So great is the diversity and variation of ENMs that one report estimated conducting traditional *in vivo* toxicologic studies on the nanomaterials currently in commerce could take more than 50 years and cost upwards of $1 billion^36^—not to mention the sheer number of test animals required for such an endeavor. So there are several robust efforts under way to develop alternative testing protocols.

In one such effort, researchers in the EPA’s ToxCast™ program are testing a small number of ENMs to see whether they are appropriate candidates for the program’s high-throughput *in vitro* assays. Preliminary results indicate they are, says ToxCast leader Keith Houck, and this fall the EPA hopes to start bulk tests in earnest, focusing on various forms of ENMs including silver and titanium dioxide nanoparticles and carbon nanotubes. The goal is to use ToxCast data to prioritize ENMs identified as hazardous for more detailed study and *in vivo* testing elsewhere.

Ultimately, asking the question “how dangerous are nanomaterials?” is likely to be fruitless, Maynard says. “There’s no answer to that because some nanoparticles are going to be safe, some of them are going to be dangerous, some of them are just going to be very different. But if you ask a very specific question, like how dangerous are titanium nanoparticles of a certain size or a certain shape and what are we going to do about it, then you’ve got something that you can begin to apply scientific principles to.”

## Making Progress

Although progress began slowly, by most accounts significant strides have been made recently in developing detection devices, advancing research methodology, and accumulating both *in vivo* and *in vitro* toxicity data—and the pace should only quicken. “There’s so much more information coming out, particularly in the last year to two years,” Walker says. “But the flip side is, it’s a huge field, so it’s always only a drop in the bucket.”

Progress can’t come fast enough for critics who accuse the federal government— the chief funder of ENM health and safety research in the United States—of selling this study area short. Fifteen different federal agencies conduct nanotechnology research, and their funding is reported through the National Nanotechnology Initiative (NNI).^37^ The NNI’s 2010 research budget totaled an estimated $1.78 billion. About 5% of that was devoted to environmental, health, and safety research, with the rest going toward things like basic research into nanomaterial behavior, research facilities, and developing nanoscale devices and systems.

While pointing out that money for safety studies has increased every year and that study findings are starting to gel, Sally Tinkle, deputy director of the NNI’s National Nanotechnology Coordination Office, concedes, “If there were more dollars we would move faster.” But Tinkle also says that, from the information accumulated so far, no acute health problems attributable to nanomaterials have been reported among workers, researchers, or consumers, although the effects of chronic exposure still require study. “It doesn’t mean we should be less vigilant or we should slow down the pace of research, but it is a little bit reassuring,” she says.

Despite all the uncertainties, many researchers express a similar sort of cautious optimism that nanomaterials will not follow the path of asbestos, polychlorinated biphenyls, and other harmful industrial agents. “I’m fairly confident that we’re not going to have an epidemic of nanodiseases in twenty to forty years, similar to the epidemic of asbestos-related diseases that we had in the twentieth century,” says Agnes Kane, a Brown University researcher whose rodent studies have shown that carbon nanotubes can produce effects very similar to those of asbestos fibers following instillation in the trachea or injection into the abdominal cavity.^38^,^39^,^40^,^41^

Maynard voices similar thoughts. “I think there is a greater chance that we’re going to see long-term environmental impacts from these materials than we are going to see shortterm consumer impacts,” he says. At the same time, he cautions, “That is informed speculation because there are so many gaps in our knowledge.”

Many researchers point to the emerging field known as green nanotechnology that is attempting to make ENMs and their production processes safer for people and the environment. They also look ahead to applications like the targeted delivery of chemotherapy drugs, tiny foodborne contaminant sensors, and advanced air- and water-filtration systems as plausible advances that could truly benefit society.

But while many critics say they are enthusiastic about some of those positive applications, they remain adamant that safety research and regulation must catch up and keep up with the technology’s proliferation. “I think we need to take a precautionary approach because we’ve learned the hard way over and over and over again,” says Hansen. “You’d think we would learn.”

## Figures and Tables

**Figure:**
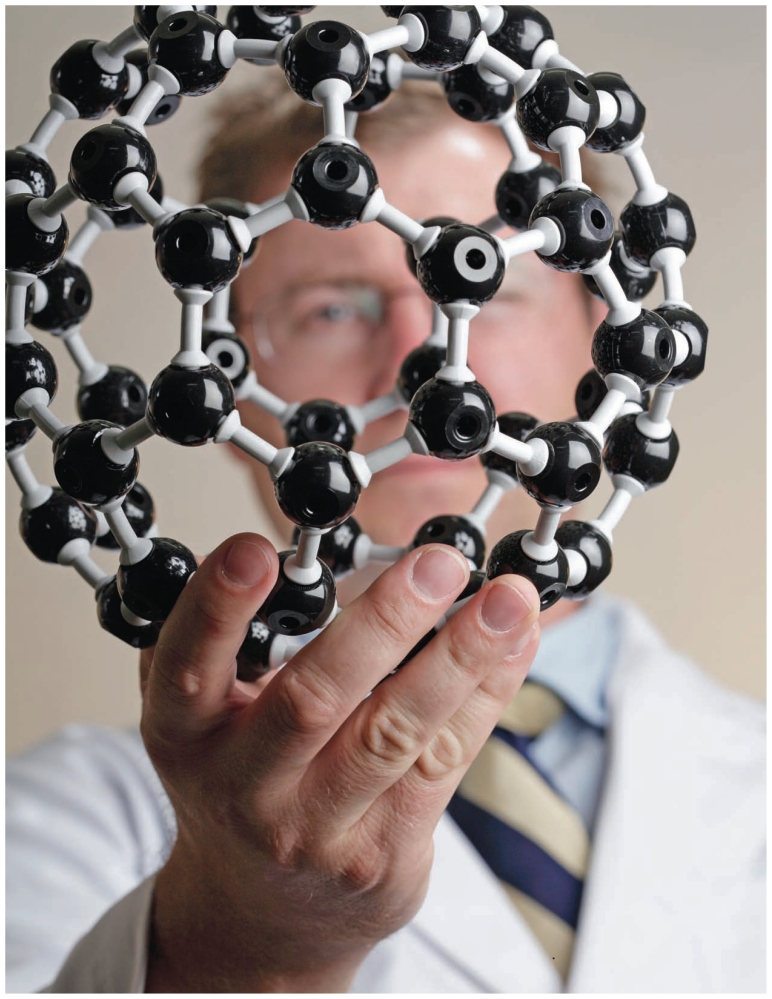
A precautionary approach to ENMs in consumer products may prevent a repeat of past episodes in which seemingly invaluable chemical innovations proceeded with little oversight and ultimately caused major health or environmental problems.

**Figure:**
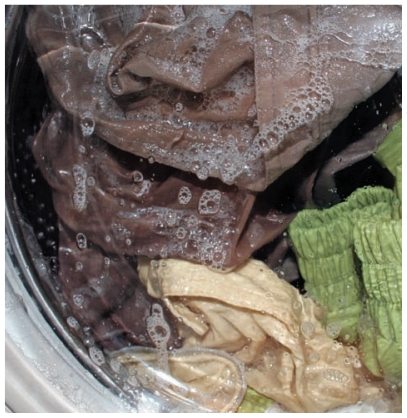
If ENMs get into the environment, new routes of exposure open up for humans (through drinking water, for example) and other organisms. Silver nanoparticles, which are used for their antimicrobial properties, have drawn research scrutiny for their environmental fate. Many researchers consider these particles quite likely to enter the aquatic environment because they can wash out of antimicrobial clothing and washing machines and into wastewater—although whether they do so in amounts large enough to matter has been subject to debate.^42^ They can also wind up in sewage sludge, which is often applied to farmland as fertilizer. Silver nanoparticles have been shown to damage cells derived from human and mammalian skin, liver, lung, brain, vascular, and reproductive tissues when evaluated *in vitro*.^43^ At high doses, they have been shown to compromise the blood–brain barrier and cause neurotoxicity in rats and mice.^5^,^44^,^45^ A 2008 University of Florida study found that both silver and copper nanoparticles can be toxic to model aquatic organisms including zebrafish, two species of water flea, and the alga *Pseudokirchneriella subcapiata*.^46^

**Figure:**
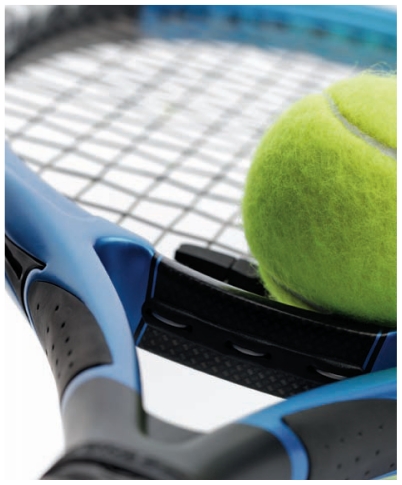
Unlike the nanomaterials in food or personal care products, which may come into direct contact with consumers’ bodies, those in many other types of goods are securely embedded in a composite matrix. Examples include bicycle parts, tennis rackets, and other sporting goods made lighter and stronger with ENMs such as carbon nanotubes. But while consumers may not be exposed in these cases, exposures and any attendant hazards are still an issue for the workers who make the goods, says Brown University researcher Agnes Kane. “We really do need to be very careful to limit exposure during the manufacturing process and the fabrication process of these materials,” Kane says. “Once they’re in composites and then used in that way, it’s less hazardous. But then we have to consider end of product life and how they would be disposed of or recycled.” As this article was going to press, the EPA announced it had awarded $5.5 million to three consortia to support innovative health and safety research on ENMs. According to an EPA press release, the grants “will help researchers determine whether certain nanomaterials can leach out of products such as paints, plastics, and fabrics when they are used or disposed of and whether they could become toxic to people and the environment.”^47^
